# Transplanted lungs and the “white plague”: A case-report and review of the literature

**DOI:** 10.1097/MD.0000000000006173

**Published:** 2017-03-31

**Authors:** Nadim Cassir, Robin Delacroix, Carine Gomez, Véronique Secq, Martine Reynaud-Gaubert, Pascal-Alexandre Thomas, Laurent Papazian, Michel Drancourt

**Affiliations:** a Aix Marseille Univ, URMITE, UM63, CNRS 7278, IRD 198, INSERM 1095, IHU Méditerranée Infection, Marseille; b APHM, Service de Pneumologie, Equipe de Transplantation pulmonaire; c APHM, Service d’Anatomo-Pathologie; d APHM, Service de Chirurgie Thoracique, Equipe de Transplantation pulmonaire; e APHM, Service de Réanimation Détresses Respiratoires et Infections Sévères, Hôpital Nord, Marseille, France.

**Keywords:** case-report, immunosuppression, lung, *Mycobacterium tuberculosis*, transplantation, tuberculosis

## Abstract

**Rationale:**

Solid organ transplant recipients, especially after lung transplantation, are at increased risk for *Mycobacterium tuberculosis* pulmonary tuberculosis due to lifelong immunosuppression.

**Patient concerns:**

A 41-year-old woman underwent a second bilateral lung transplantation that was complicated by fatal pulmonary tuberculosis.

**Diagnoses:**

Histological examination of a lung biopsy performed 6 weeks after retransplantation revealed a caseating granuloma and necrosis. Acid-fast bacilli were identified as rifampicin-susceptible *M. tuberculosis* by real-time polymerase chain reaction (PCR), confirmed by culture 2 weeks later.

**Interventions:**

Our investigation led us to highly suspect that the transplanted lungs were the source of *M. tuberculosis* transmission.

**Lessons:**

In order to optimize diagnosis and treatment for lung recipients with latent or active tuberculosis, regular assessment of lower respiratory samples for *M. tuberculosis*, particularly during the 12-month period posttransplant should be implemented. Regarding donor-derived transmission, screening donor grafts with latent tuberculosis by *M. tuberculosis* real-time PCR in lymphoid and adipose tissues is an option that should be considered.

## 1 Introduction

Solid organ transplant (SOT) recipients are at increased risk for *Mycobacterium tuberculosis* pulmonary tuberculosis due to lifelong immunosuppression.^[1]^ In low tuberculosis-prevalence regions, the frequency of pulmonary tuberculosis in SOT recipients varies from 1.2% to 6.5%.^[2]^ In this population, diagnosis delay, treatment-related toxicities, and drug interactions complicate the management of tuberculosis, leading to a up to 30% mortality.^[1]^ Posttransplantation tuberculosis may result from reactivating latent tuberculosis in the recipient or transmission of *M. tuberculosis* from a contagious person or from the transplant.^[1]^ Risk for pulmonary tuberculosis is greater for lung transplant receivers compared with other SOT recipients.^[3]^ In this population, the onset of pulmonary tuberculosis varies from 1 day to 12 months after lung transplantation.^[4,5]^

In our hospital, diagnosing deadly pulmonary tuberculosis 8 weeks after bilateral lung transplantation led to investigate the source of *M. tuberculosis*. This case is reported anonymously in agreement with the advice n°2016–024 of the Méditerranée Infection Institute Ethics Committee.

## 2 Case report

**Figure 1:**
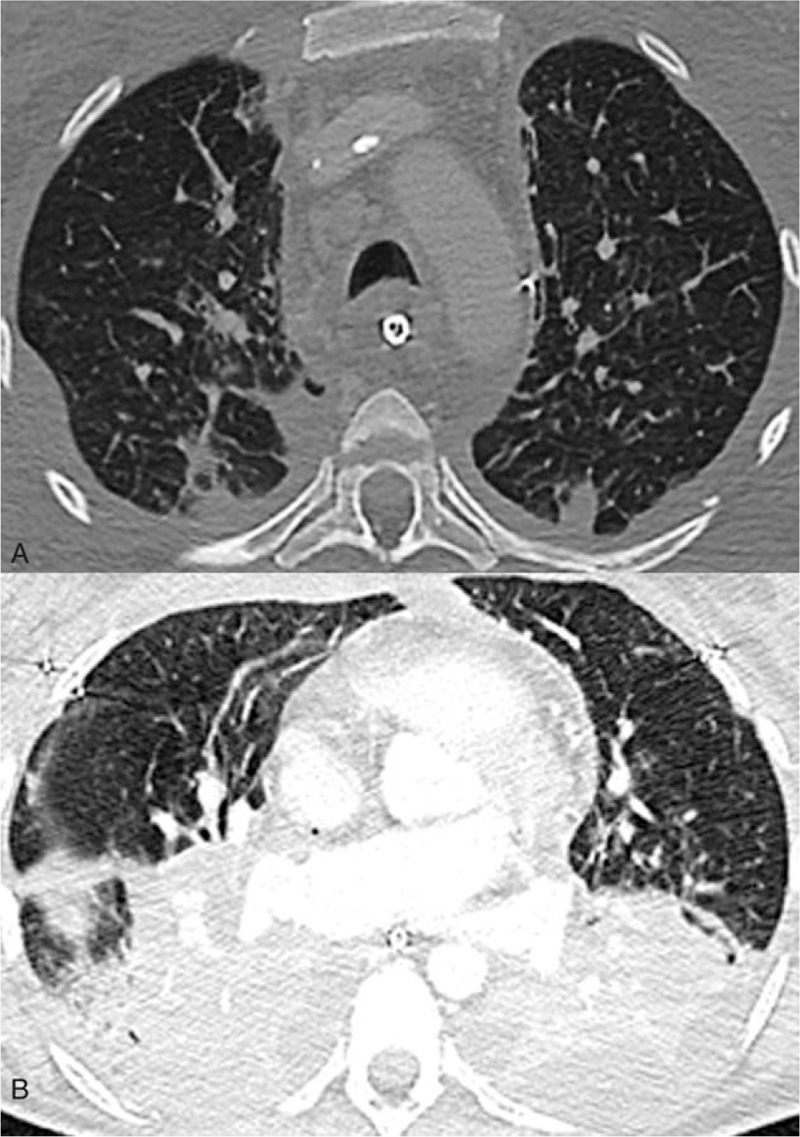
Chest computed-tomography obtained on day 68 after transplantation. A: Bilateral lung parenchymal nodules with cavity in right lower lobe apical segment. B: Bilateral pleural effusion with nodule in posterior segment of right lower lobe.

A 41-year-old Caucasian woman underwent a primary double lung transplantation for cystic fibrosis in 2006. Her medical history was otherwise unremarkable and the patient had no known history of pulmonary tuberculosis or tuberculosis contact. On December 2015, she underwent retransplantation for chronic lung allograft dysfunction. During the month preceding retransplantation, 4 sputum specimens remained negative for acid-fast bacilli and specific *M. tuberculosis* culture and real-time polymerase chain reaction (PCR) testing. On postoperative day 42, deterioration of her respiratory status prompted a chest-computerized tomography (CT) scan revealing sub-centimeter bilateral nodules primarily located in the apical posterior lobes and a bilateral pleural effusion (Fig. 1). The same day, a bronchoalveolar lavage (BAL) yielded a positive real-time PCR for rifampicin-susceptible *M. tuberculosis*, confirmed by culture on postoperative day 62. Tuberculin skin test (TST) or interferon-γ release assay (IGRA) test were not performed. All the BALs performed on postoperative period yielded no other pathogen except for the one performed on day 60 that cultured *Pseudomonas aeruginosa*; the adjunctive antibiotic therapy was imipenem-cilastatin, 3 g/d. Histological examination of a lung biopsy performed 6 weeks after retransplantation revealed a caseating granuloma and necrosis. Acid-fast bacilli were identified as rifampicin-susceptible *M. tuberculosis* by real-time PCR. On postoperative day 65, the patient's status worsened with severe hypoxemia, shock unresponsive to high dose cathecolamines, and multiorgan failure. The patient died on postoperative day 70, despite treatment combining isoniazid, rifampicin, ethambutol, and pyrazinamide. Retrospective real-time PCR testing of the explanted lung and BALs performed on postoperative days 1, 7, and 21 remained negative.

The organ donor died of posttraumatic intracerebral hemorrhage. He was a 47-year-old man with no history of lung disease or risk factors for tuberculosis other than chronic alcohol use and smoking. TST results were not available. During hospitalization, a lung CT-scan showed no signs of active or previous tuberculosis and no TST or IGRA test results were available. Routine cultures of per-transplantation right lung biopsy yielded *Candida albicans*. Retrospective *M. tuberculosis* real-time PCR yielded negative results on the left and right donor-lung biopsies. Both kidneys from the same donor were transplanted into 2 other recipients. Six months after transplantation, neither of the kidney recipients had developed any signs or symptoms suggestive of active tuberculosis.

## 3 Discussion

Several lines of evidence indicate that the transplanted lungs were the source of fatal pulmonary tuberculosis in the patient who underwent a second bilateral lung transplantation. During her 9-year history of her first bilateral lung transplant, the recipient had no known history of tuberculosis. In the month prior to second transplantation, she presented no clinical, CT-scan, or microbiological evidence of pulmonary tuberculosis. During regular monitoring, the first positive respiratory sample tested positive for *M. tuberculosis* was obtained 42 days after the second transplantation, while immunosuppressive therapy had been administered for 9 years following the first transplantation. Investigations found no evidence of a new infection posttransplant *via* healthcare-associated cross-transmission that could otherwise have explained this case. No case of active tuberculosis infection was diagnosed among her relatives, other patients or healthcare workers during the 3-month pretransplant period and the posttransplant stay in the thoracic surgery ward or intensive care unit.

A donor-to-recipient lung transmission was suspected in 15 cases of pulmonary tuberculosis in lung transplant recipients since 1990 (Table 1 ).^[5–15]^ It was conclusive in only 1 case reporting a 14-year-old girl with chronic bronchiectasis who was TST-negative before transplantation.^[15]^ She received a bilateral lung transplant from a 51-year-old man born in the Philippines with a solitary pulmonary nodule that was found on perioperative palpation. Histologic analysis of this nodule indicated a caseating granuloma and necrosis with positive AFB staining and the recipient BAL performed on postoperative day 5 was positive for *M. tuberculosis* by PCR and culture. Early initiation of antituberculosis treatment and the omission of induction immunosuppressive therapy led to a favorable outcome.

**Table 1:**
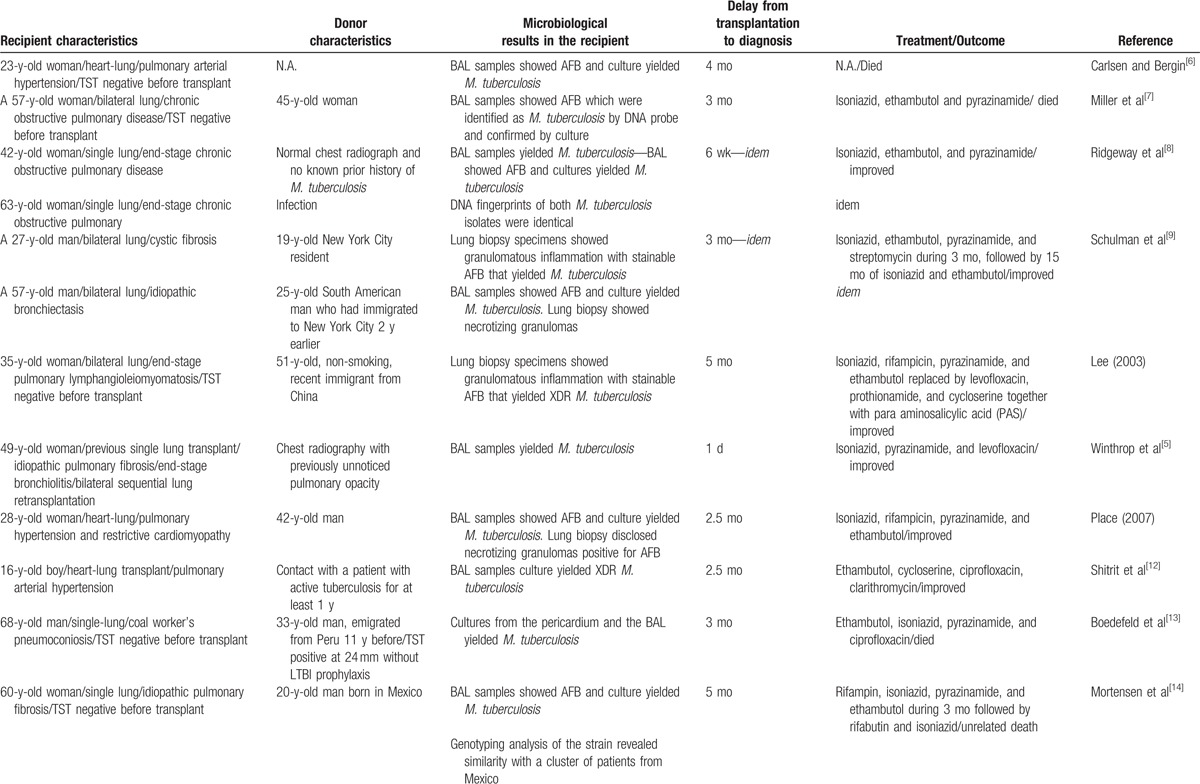
Cases of donor-derived pulmonary tuberculosis after lung transplantation since 1990.

**Table 1 (Continued):**
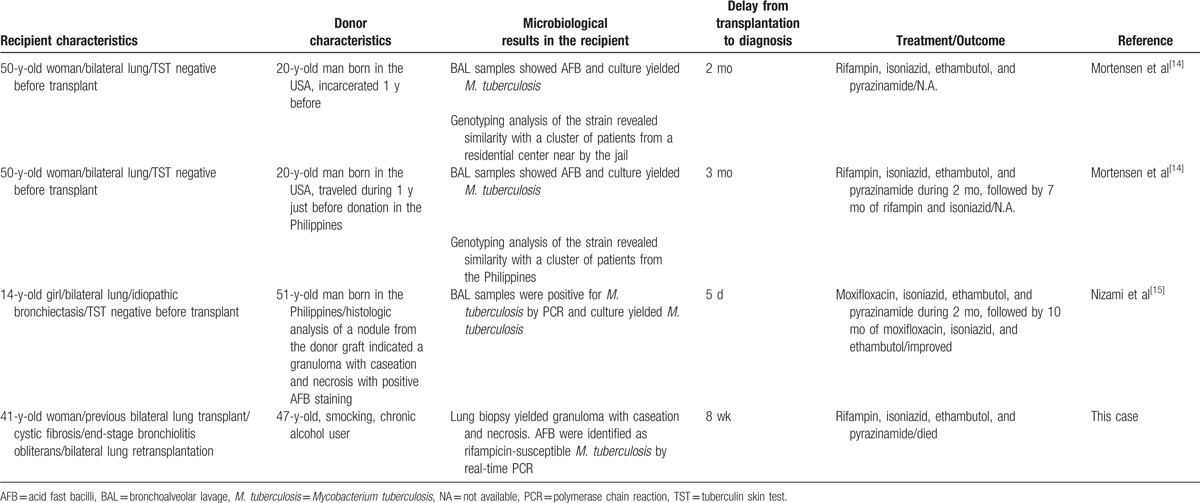
Cases of donor-derived pulmonary tuberculosis after lung transplantation since 1990.

Current US and European guidelines recommend routine screening and treatment for latent tuberculosis infection (LTBI) in lung recipients but there is no controlled trial.^[1,16]^ Assessing LTBI includes reviewing epidemiologic risk factors, chest radiography and a TST and/or IGRA. However, TSTs and IGRAs are less sensitive in immunosuppressed and/or critically ill patients than in the general population and they do not differentiate LTBI from active tuberculosis.^[17]^

There is no controlled trial to support specific recommendations regarding lung donors.^[1]^ One option would be to screen lungs just before or at the time of transplantation as early antituberculous treatment and immunosuppression optimization are essential to successfully treat lung recipients with active tuberculosis.^[15]^ In the case reported here, negative retrospective detection was obtained on fixed rather than fresh biopsies. TST or IGRA testing in deceased donors is difficult to perform and to interpret.^[14]^ Because *M. tuberculosis* DNA is detected in lymphoid and adipose tissues surrounding the lungs in LTBI patients, the cost-effectiveness of rapid real-time PCR testing of the donor lungs has to be evaluated in various tuberculosis prevalence settings.

## 4 Conclusion

Given the substantial morbidity and mortality associated with active tuberculosis in lung recipients, it is crucial to come up with an early diagnosis for those with latent or active tuberculosis in order to optimize their treatment. Regular assessment of lower respiratory samples for *M. tuberculosis*, particularly during the 12-month period posttransplant should be implemented. Regarding donor-derived transmission, screening donor grafts with LTBI by *M. tuberculosis* real-time PCR in lymphoid and adipose tissues is an option that should be considered.
